# Primary tuberculoma of the liver: a case report and literature review

**DOI:** 10.11604/pamj.2014.19.321.5107

**Published:** 2014-11-26

**Authors:** Ghizlane Kharrasse, Mehdi Soufi, Hayat Berekhli, Hidaya Intissar, Mohammed Bouziane, Zahi Ismaili

**Affiliations:** 1 Department of Gastroenterology, Faculty of Medicine Oujda, University Mohammed first, Oujda, Morocco; 2 Department of Digestive Surgery, Faculty of Medicine Oujda, University Mohammed first, Oujda, Morocco; 3 Department of anesthesiology; 4 Department of nephrology, Faculty of Medicine Oujda, University Mohammed first, Oujda, Morocco

**Keywords:** Tuberculosis, liver, tuberculome, antitubercular therapy

## Abstract

We report the case of an immunocompetent patient with an isolated tuberculoma of the liver, which was diagnosed by percutaneous US-guided liver biopsy. The patient received an antitubercular therapy, and there has been no relapse to date.

## Introduction

Hepatic tuberculosis is an uncommon form of extrapulmonary tuberculosis. It is usually a disseminated disease associated with miliary tuberculosis, which is one of the most characteristic manifestations of tuberculosis (TB). Localized tuberculosis of the liver in the form of macronodular tuberculoma or an abscess is rare [1]. However, because of the increasing incidence of pulmonary tuberculosis, clinicians should be aware of the possibility of tuberculous infection in all patients who have non-resolving liver abscesses, particularly in regions with high prevalence, such as Morocco. We describe a rare case of tuberculous liver with no evidence of lung or ileocecal involvement [2].

## Patient and observation

A 44-year-old man with a history of benign gastroesophageal reflux disease (GERD), with irregular use of IPP, visited the emergency department with the chief complaint of intermittent fever for 3 months. Body weight loss (11 kg/month), abdominal fullness, poor appetite, and general malaise were also observed. He had no significant history of tuberculosis, and no relevant familial history. On direct questioning he admitted to have night sweats and abdominal pain. The patient said that he had received outpatient treatment followed by oral antibiotics. Unfortunately, his condition worsened with intermittent chills and body weight loss, so he was referred to our hospital. On admission, we found a mildly cachetic man. No jaundice was noted by examination of the palpebral or bulbar conjunctiva, but he was found to be pyrexial (38.7°C on admission). His heart rate was 91 beats/min, blood pressure was 135/89 mmHg, respiratory rate was 18 breaths/min, and oxygen saturation was 100% under normal conditions. Physical examination revealed mild right hypochondrial knocking pain with right hepatomegaly. Palpation did not reveal any abnormalities in the gallbladder or spleen, no abnormality was noted by chest percussion and there was no lymphadenopathy.

**Figure 1:**
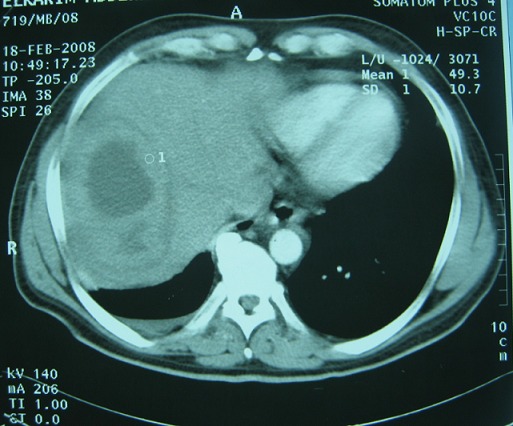
CT scan showing the hepatic tuberculoma

**Figure 2:**
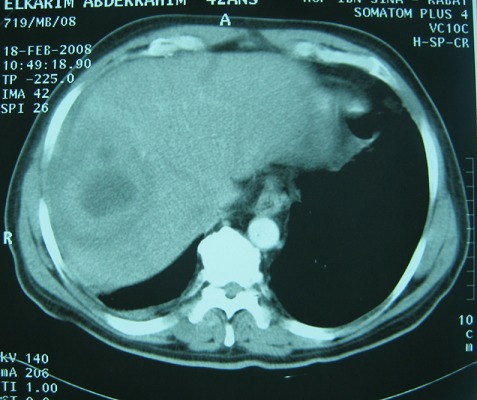
CT scan showing a 7 cm low-density lesion with central necrosis

The results of laboratory studies showed the following: an elevated C reactive protein (CRP) level of 152 (normal less than 10 mg/L). a mildly elevated erythrocyte sedimentation rate (ESR), and hyponatremia at 121mEq/L (Normal serum sodium levels are between approximately 135 and 145 mEq/L); a normal potassium value at 4.16 mEq/L (normal range between 3.5 and 5.0 mEq/L); white blood cell count (WBC) of 8500/mL (normal range, 3500-9100/mL); hemoglobin, 10.9 g/dL (normal range, 13.5-18 g/dL), platelet count, 280,000/μL (normal range, 157,000-377,000/ μL); aspartate aminotransferase (AST), 18 IU/L (normal range, 11-39 IU/L); alanine aminotransferase (ALT), 25 IU/L (normal range, 4-38 IU/L); total bilirubin, 2.2 mg/dL (normal range, 0.2-1.0 mg/dL); elevated creatinine, 3.2 mg/dL (normal range, 0.6-1.5 mg/dL); blood urea nitrogen (BUN), 21 mg/dL (normal range, 9-23 mg/dL). Prothrombin time was within normal limits. Further evaluations, including tests for human immunodeficiency virus, carcinoembryonic antigen, cancer antigen 19-9, α-fetoprotein, prostate-specific antigen, serologic diagnosis of hydatid disease and amoebic hemagglutination were all negative. Several sets of blood and sputum cultures were negative for bacteria, fungus, and acid-fast bacilli. Abdominal ultrasound revealed marked hepatomegaly with a tumor with an unclear border in the VIII subsegment of the liver consisting partially of hypoechoic mass, with the hyperechoic area measuring 7 cm. No ascites or adenopathy was seen. Similarly, Abdominal computed tomography (CT) revealed a low-density lesion approximately 7 cm in diameter at the SVIII segment, showing various fat concentrations. Chest X-ray examination was normal (Figure 1, Figure 2). Therefore, we scheduled a liver biopsy in order to rule out malignancy and to have a definitive diagnosis. The direct appreciation of the exudates did not reveal bacteria and histological study suggested an inflammatory pseudotumor with Langerhans giant cells and without caseous necrosis. Anti-TB medications including isoniazid, rifampin, ethambutol, and pyrazinamide were administered accordingly. The fever subsided within 1 week, and the patient's appetite was restored gradually. The patient completed the 6-month course of medication (2 months with isoniazid, rifampin, ethambutol, and pyrazinamide, followed by 4 months with isoniazid, rifampin, and ethambutol) without severe adverse events. WBC count, CRP, and ESR all returned to normal levels. The patient's appetite and body weight were restored to previous levels. The follow-up abdominal CT scan revealed that the lesion at the S8 segment had decreased in size after 4 months of treatment and disappeared after one year of follow up. Follow-up was continued for six years, and the patient is still healthy.

## Discussion

Tuberculosis is a monumental health problem in the developing world and it remains a healthcare challenge in the developed world owing to immigration from endemic areas, increased prevalence of immunosuppression, and emergence of multidrug- and extensively drug-resistant strains of Mycobacterium tuberculosis [3]. Abdominal TB is a rare manifestation among various forms of extrapulmonary TB. It can develop from (a) reactivation of a dormant focus in the abdomen following haematogenous spread during an earlier primary infection, (b) haematogenous or lymphatic spread from current active tuberculosis, (c) ingestion of the pathogen, or (d) by direct extension from adjacent involved tissues [4].

Liver TB is considered very rare among abdominal TB patients. Usually it is associated with foci of infection in the lungs or gastrointestinal tract. TB spreads to the liver through the hepatic artery, the portal system and the lymphatic system. Liver TB commonly involves the hepatic parenchyma and sometimes the biliary tree. The presentations of liver TB are divided into three types: Miliary hepatic TB which is haematogenously disseminated from the lungs via hepatic artery, and it is considerered the most common type of hepatic TB [5]; Biliary tract TB which may have varied presentations but usually presents as a triad of fever, jaundice, and hepatic calcifications. Jaundice is due to extrahepatic or intrahepatic strictures, adenopathy, or hepatolithiasis [6].

Nodular hepatic TB as in our case, which is most unusual and almost all publications have been single case reports. The localized form of tuberculosis of the liver, termed also a tuberculoma, was first described over a century ago by Bristowe (1858) who found the liver to be involved in 12 out of 167 cases of tuberculous ulceration of the intestines [7]. Thus, Tuberculoma and tuberculous liver abscesses are uncommon manifestations of hepatic TB. When they appear as discrete nodules, diagnosis can be difficult. The clinical presentation is not specific and a high degree of clinical suspicion is required to diagnose the entity. While it can be easily managed medically, if, not treated it can lead to hepatic failure and ultimately death [8]. The most common symptoms are right upper quadrant pain, fever, anorexia, and weight loss. Hepatomegaly may be found with an increase in alkaline phosphatase, and elevation of transaminases may be present in two-thirds of the cases. Anemia and elevated erythrocyte sedimentation rate (ESR) are often seen [9]. Imaging can play an important role in the detection, characterization, and management of liver tuberculosis [4]. Ultrasound is considered the imaging modality of choice for initial screening.

On ultrasonography, hepatic tuberculoma usually shows well marginated hypoechoic lesion with or without calcifications [8], and hyperechoic lesion is rarely seen [10]. The computed tomographic (CT) appearance of this lesion will vary depending on its evolutionary stage (solid, necrotic or fibrous). The LT is typically a well-circumscribed lesion with a moderate peripheral enhancement. The canalicular form is manifested by expansion of intrahepatic bile ducts with calcifications along their walls [11]. The differential diagnosis of micronodular hepatic tuberculosis includes metastases, lymphoma, leukemia, sarcoidosis, and fungal infection. The macronodular form seen as large lesions with peripheral rim enhancement and central low attenuation on CT may appear identical to pyogenic abscess, metastases, and primary liver tumors like hepatocellular carcinoma and cholangiocarcinoma. Isolated tubercular abscess mimics pyogenic liver abscess on imaging [11, 12]. Knowledge of the wide spectrum of CT appearances of abdominal tuberculosis should alert the radiologist to consider its diagnosis, especially among high-risk groups of patients, however the definitive diagnosis of this disease is conventionally made by acid-fast staining of clinical isolates followed by culture or histological examination of the tissue specimens for evidence of caseation with granuloma [4].

Otherwise, the rate of accurate pretreatment diagnosis by guided percutaneous liver biopsy has been reported to be low, and the presence of tubercle bacilli in the biopsy sample is rarely reported. Thus, the reliability of needle biopsy as a diagnostic method seems uncertain [10]. However, the use of polymerase chain reaction to directly detect the presence of *Mycobacterium tuberculosis* is increasing and may improve sensitivity rates [13]. For most authors the diagnosis can be reached based either on the presence of hepatic granulomas associated with documented tuberculosis in another organ, particularly the lungs, or when the clinical symptoms and radiological examination evidence regress after starting up antituberculous treatment, particularly if the initial antibiotic therapy failed [14]. Hepatic tuberculosis is treated like any other extrapulmonary tuberculosis lesion. Chemotherapy with standard anti-tuberculosis drugs remains the cornerstone of treatment. This is true for both diffuse as well as the local forms of the disease. In general, a 6- to 9-months regimen 2 months of isoniazid, rifampin, pyrazinamide, and ethambutol followed by 4-7 months of isoniazid and rifampin) is the recommended treatment for extrapulmonary tuberculosis [6]. Cumulative mortality for hepatic tuberculosis ranges between 15% and 42% [2, 9]. The factors associated with adverse prognosis are: age < 20 years, miliary tuberculosis, concurrent steroid therapy, AIDS, cachexia, associated cirrhosis and liver failure. Drug induced hepatotoxicity is not mentioned in most reports of liver tuberculosis even with the widespread use of Rifampicin. In all probability, this may be due to the small number of patients with hepatic tuberculosis rather than a true absence of hepatotoxicity. In addition to chemotherapy, anecdotal reports of successful percutaneous drainage of tuberculous liver abscesses have been made [15]. Ultrasound and / or CT can be proposed in assessing therapeutic response to antitubercular treatment [16].

## Conclusion

Localized tuberculosis of the liver in the form of macronodular tuberculoma or an abscess is rare. However, because of the increasing incidence of pulmonary tuberculosis, clinicians should be aware of the possibility of tuberculous infection in all patients who have non-resolving liver abscesses, particularly in regions with high prevalence, such as Morocco. Since clinical features are protean and mimic neoplastic and infective hepatic disorders, the diagnosis requires a high index of suspicion. We suggest that tuberculous liver abscess should be considered in patients not showing typical features or who fail to respond to antibiotics. The demonstration of granulomas on liver biopsy remains the most sensitive diagnostic procedure, As for other forms of extra pulmonary tuberculosis, hepatic tuberculosis is a potentially curable disease. Good results have been obtained with four drug regimens without any added risk of hepatotoxicity as in our case.
